# What endocrinologists can do to prevent cardiovascular complications in adults with Prader-Willi syndrome: Lessons from a case series

**DOI:** 10.3389/fendo.2023.1145066

**Published:** 2023-03-24

**Authors:** Karlijn Pellikaan, Paula M. H. van Weijen, Anna G. W. Rosenberg, Franciska M. E. Hoekstra, Michiel Vermaak, Peter H. N. Oomen, Aart J. van der Lely, Judith A. A. E. Cuypers, Laura C. G. de Graaff

**Affiliations:** ^1^ Department of Internal Medicine, Division of Endocrinology, Erasmus MC, University Medical Center Rotterdam, Rotterdam, Netherlands; ^2^ Center for Adults with Rare Genetic Syndromes, Department of Internal Medicine, Division of Endocrinology, Erasmus MC, University Medical Center Rotterdam, Rotterdam, Netherlands; ^3^ Dutch Center of Reference for Prader-Willi Syndrome, Rotterdam, Netherlands; ^4^ Academic Centre for Growth Disorders, Erasmus MC, University Medical Center Rotterdam, Rotterdam, Netherlands; ^5^ Department of Internal Medicine, Reinier de Graaf Hospital, Delft, Netherlands; ^6^ Novicare, Best, Netherlands; ^7^ Department of Internal Medicine, Medical Center Leeuwarden, Leeuwarden, Netherlands; ^8^ Department of Cardiology, Erasmus MC, University Medical Center Rotterdam, Rotterdam, Netherlands

**Keywords:** Prader-Willi syndrome, cardiovascular system, comorbidity, heart failure, cardiovascular abnormalities

## Abstract

**Context:**

Prader-Willi syndrome (PWS) is a complex rare genetic syndrome. Mortality in patients with PWS is 3% per year. In nearly half of the patients, the cause of death is of cardiopulmonary origin. Prevention, diagnosis and treatment of cardiovascular (CV) disease in PWS adults is complicated by the behavioral phenotype, reduced ability to express physical complaints, high pain threshold and obesity.

**Objective:**

To describe the challenges in prevention, diagnosis and treatment of CV disease in PWS adults, in order to increase awareness and improve medical care.

**Methods:**

Retrospective study of medical records of adults visiting the Dutch PWS reference center.

**Results:**

We describe the challenges encountered during diagnosis and treatment of four PWS adults with heart failure. All had pre-existent peripheral edema. CV risk factors in these patients were obesity (n=4), type 2 diabetes mellitus (n=2), hypertension (n=2), hypogonadism (n=3) and sleep apnea (n=2). Remarkably, all patients were younger than 40 years during their first cardiac decompensation. All patients presented with progressive shortness of breath and/or orthopnea and progressive pitting edema. In 117 controls with PWS without CV problems, 31% had leg edema.

**Conclusion:**

Diagnosing CV problems in PWS adults is challenging. Peripheral edema is common in PWS adults without CV morbidity, which makes edema in general a poor marker for heart failure. However, when edema is of the pitting kind and progressive, this is a strong predictor of cardiac decompensation. We provide practical recommendations for diagnosing and treating CV problems in this vulnerable patient population.

## Introduction

Prader-Willi syndrome (PWS) is a complex genetic disorder caused by the loss of function of a cluster of paternally expressed genes on chromosome 15q11.2-q13. It occurs in 1:15.000 – 25.000 live births (1). PWS can result from a paternal deletion (65-75%), a maternal uniparental disomy 15 (mUPD, 20-30%), an imprinting center defect (ICD, 1-3%) or a paternal chromosomal translocation (0.1%) (2, 3). During infancy, patients with PWS often have muscular hypotonia, low muscle mass, feeding difficulties, failure to thrive and delayed development. During childhood, most patients develop an insatiable appetite, often leading to obesity. Patients with PWS have an abnormal body compositon with a high fat mass and low muscle mass (4, 5). Additionally, patients with PWS have hypothalamic dysfunction resulting in pituitary hormone deficiencies, abnormal temperature regulation and inadequate pain registration (6–9).

Mortality in patients with PWS is as high as 3% per year (10, 11). In nearly half of the patients, the cause of death is of cardiopulmonary origin and three-quarters of deaths are unexpected (10, 12). Cardiovascular (CV) abnormalities can occur already early in life (13) and patients with PWS have an increased risk to develop CV disease at a young age (10, 14–16). As previously described (17), this increased CV risk is caused by a complex interplay between somatic and behavioral aspects of the syndrome. Musculoskeletal problems associated with the syndrome (i.e. scoliosis and foot problems), hypotonia and pituitary hormone deficiencies can lead to poor exercise tolerance, which can be further aggravated by behavioral challenges. This exercise intolerance combined with hyperphagia, can lead to an increase in body fat and decrease in lean body mass. This leads to a low basal rest metabolism, which further deteriorates body composition. Increased body fat eventually leads to an increased prevalence of CV risk factors like type 2 diabetes mellitus (DM2), hypertension, hypercholesterolemia and sleep apnea (10, 18–24).

Besides abnormal body composition, appetite regulating hormones like ghrelin and leptin can also affect the CV system. PWS is associated with high ghrelin levels and an elevated acylated ghrelin/unacylated ghrelin (AG/UAG) ratio (25). While high levels of both AG and UAG seem to have protective CV effects (26–28), high ghrelin levels could cause weight gain and glucose intolerance (29, 30). Another key player in appetite regulation, leptin, has been associated with the presence, severity, extent and lesion complexity of coronary atherosclerosis (31). Leptin levels in non-obese PWS males are nearly five times higher than in non-obese control males (32). However, the interplay between leptin levels, obesity and CV pathology in PWS has not been investigated.

Apart from the complex etiology, the diagnostic trajectory of CV disease in adults with PWS is also complex. Physicians usually rely on common symptoms of heart disease, that are reliable indicators of cardiac problems in the general population, such as chest pain, orthopnoea, shortness of breath, decreased exercise tolerance, palpitations, fatigue and peripheral edema (33, 34). However, in PWS, some of these symptoms are less reliable. Chest pain can be easily missed due to the high pain threshold in PWS and many PWS adults lack the verbal skills to express complex physical complaints like orthopnoea and palpitations. Physical signs like decreased exercise tolerance and peripheral edema are already common in adults with PWS without CV disease (17, 22, 23) and are therefore unreliable parameters. This combination of factors can easily lead to diagnostic delay (3, 6, 7). The diagnostic process can be further hindered by obesity, which complicates the interpretation of transthoracic cardiac ultrasound (35) and can lead to false-normal NT-proBNP values (36).

In the current case series, we describe the challenges that occurred during the diagnostic trajectory and treatment of four adults with PWS and CV problems. Moreover, we compare clinical characteristics between patients with and without CV events. Based on this comparison and literature data, we provide clinical recommendations for the diagnosis and treatment of CV disease in adults with PWS.

## Methods

This study was approved by the Medical Ethics Committee of the Erasmus University Medical Center, Rotterdam, the Netherlands. In this retrospective study, we reviewed medical files of adults with PWS who underwent our routine systematic health screening at the multidisciplinary outpatient clinic of the Center for Adults with rare genetic syndromes at the Erasmus University Medical Center, Rotterdam, the Netherlands between 2015 and 2022. This systematic screening consists of a structured interview, a complete physical examination, a medical questionnaire, a review of the medical file and biochemical measurements, as described previously (17). Biochemical measurements that were routinely measured were: low density lipoprotein (LDL)-cholesterol, nonfasting glucose, hemoglobin A1c, thyroid-stimulating hormone, free thyroxine, luteinizing hormone, follicle-stimulating hormone, estradiol or testosterone, sex hormone binding globulin, aspartate transaminase, alanine transaminase, alkaline phosphatase, gamma glutamyl transpeptidase, total bilirubin, lactate dehydrogenase, urea, creatinine, estimated glomerular filtration rate, hemoglobin, hematocrit, mean corpuscular volume, leukocytes, thrombocytes and 25-hydroxyvitamin D.

We systematically assessed symptoms of CV disease like orthopnea, dyspnea, nocturia, swollen legs and chest pain. During physical examination the presence of edema was objectified and cardiac auscultation was performed. Additional cardiac diagnostics, like NT-proBNP, chest X-ray, cardiac ultrasound or electrocardiogram (ECG) were not performed routinely, but only if CV problems were suspected.

We describe four adults with PWS who had suffered a CV event in their medical history or who developed CV problems while under treatment at the outpatient clinic of our center. We compare these patients to 117 control patients with PWS without known CV events.

### Data analysis

Descriptive statistics for continuous variables are reported as median [interquartile range (IQR)]. For dichotomous variables we display the number and the percentage of people, n (%).

## Results

We describe four patients with CV events, all with manifestations of heart failure, of whom detailed clinical information was available.

### Patient 1

A 39-year-old male was hospitalized with severe dyspnea and weight gain. Physical examination revealed orthopnea, bronchospasm, severe pitting edema up to his chest and generalized rhonchi. He had severe tachypnea with up to 40 breaths per minute. NT-proBNP was normal (20 pmol/L). Based on the acute clinical presentation, pulmonary congestion was suspected. The ECG showed no abnormalities. Chest X-ray was normal (i.e. no signs of pulmonary congestion), besides a slightly blurred left heart margin. Intravenous diuretics were started to reduce edema and beta-agonist inhalers were started to reduce bronchospasm. Within 48 hours, he had lost three kilograms and his respiration frequency had returned to normal (16 breaths per minute). Afterwards, cardiac ultrasound showed normal systolic left ventricular function and no signs of significant diastolic dysfunction. Right ventricular pressure was increased. Although computed tomography angiography (CTA) of the coronary arteries did not reveal any significant obstructions, the calcium score was 146 (> 90^th^ percentile), which indicated the presence of coronary sclerosis. He was prescribed atorvastatine and reduced his cigarette use from 75 to 21 cigarettes per week.

CV risk factors included morbid obesity (body mass index (BMI) 45 kg/m^2^), heavy smoking, hypertension and dyslipidemia. Another risk factor was hypogonadism (37), which was untreated as testosterone therapy had caused behavioral challenges. Detailed analysis of additional CV risk factors revealed frequent hypoglycemias (38). The patient used insulin, which he administered himself. It turned out that he injected himself with too much insulin, in order to induce hypoglycemia and receive extra food. Finally, sleep apnea (apnea-hypopnea index (AHI) of 15) was also present. To reduce pulmonary resistance and optimize cardiac function, continuous positive airway pressure (CPAP) was started.

### Patient 2

A 37-year-old female was hospitalized for analysis and treatment of generalized edema, with clinical suspicion of heart failure. Nephrogenic causes of edema, like nephrotic syndrome, had been excluded. CV risk factors included a family history with CV disease, DM2, morbid obesity and hyperlipidemia. ECG showed negative T waves in V1-3, indicating right ventricular hypertrophy. Cardiac ultrasound showed tricuspid valve insufficiency with signs of elevated right ventricular pressure and a dilated vena cava inferior, but transthoracic ultrasound quality was poor due to obesity. It was hypothesized that she had pulmonary hypertension caused by morbid obesity (BMI 44 kg/m^2^) and severe sleep apnea (AHI of 59). CPAP was initiated, which led to recompensation. Three months later, she was hospitalized again. An upper respiratory tract infection had caused an increase of her pre-existent pulmonary hypertension, which caused decompensated right heart failure. After a short stay in the hospital, she could be dismissed, but returned half a year later. Then, she admitted that she had discontinued CPAP. Insufficient surveillance in her residential facility had led to nonadherence to CPAP. She had also gained a lot of weight, due to insufficient external food control. She had access to her own debit card, which she used to buy food.

### Patient 3

A 29-year-old female with hypogonadism and central hypothyroidism was hospitalized because of orthopnea, progressive exercise-related shortness of breath, hyponatremia (134 mmol/L) and progressive pitting edema. NT-proBNP was increased (99 pmol/L). Chest X-ray showed an enlarged heart. During her stay at the hospital, she developed severe epileptic seizures which eventually required intubation and transfer to the intensive care unit (ICU). Magnetic resonance imaging (MRI) and electroencephalography (EEG) did not show any abnormalities. A year earlier, she had undergone cardiac evaluation because of peripheral edema. At that time, ECG did not show any signs of ischemia and cardiac ultrasound came back normal. However, retrospectively, interpretation at that time was probably already hindered by her morbid obesity. A new cardiac ultrasound, performed at the ICU, showed a normal systolic left ventricle function (LVEF 55%), but a dilated right ventricle and tricuspid valve insufficiency. This was probably the result of right heart failure due to pulmonary hypertension, resulting from restrictive lung function caused by scoliosis and morbid obesity (BMI 48 kg/m^2^). The patient was treated with intravenous diuretics and fluid and salt restriction, after which she recompensated. After 3 weeks she was sent home with oxygen therapy.

### Patient 4

A 32-year-old male presented for the first time at our outpatient clinic. He had progressive exercise-related shortness of breath, extreme peripheral edema with blisters and weight gain of 20 kilograms in one month. He had morbid obesity (BMI 53 kg/m^2^). At physical examination, his oxygen saturation was 80%. Initially, he refused physical examination and venipuncture. ECG showed left axis cardiac deviation and a right bundle branch block, but no signs of acute ischemia. Chest X-ray showed an enlarged heart and hilar enlargement. He was admitted to the hospital for treatment of his congestive heart failure, but refused medication, oxygen and other medical interventions. As he refused medical treatment without being able to understand the consequences, he was considered to be a danger to himself. The medical staff tried to arrange the legal documents needed for involuntary commitment. However, his aggressive behavior made it impossible to keep him on the ward and the patient left the hospital. As the stress of forced hospitalization and forced treatment was expected to further aggravate his cardiac problems, it was decided to try to treat him in the residential home where he lived. He was treated with oral diuretics (bumetanide) and a salt and water restriction. Eventually, he took the prescribed medication for two days and agreed to undergo venipuncture, which showed an increased NT-proBNP (230 pmol/L). The physician for intellectual disabilities (ID physician) reported that, after an initial weight loss of 3 kilograms, he had gradually increased in weight again. The patient adhered less and less to his salt and water restriction. His hyperphagia, aggressive behavior and the complex psychosocial situation made it impossible for his physician and caregivers to improve adherence to treatment. Eventually, he ate a large amount of salty food and spent two nights drinking water in his shower. He was readmitted to the hospital due to shortness of breath, where he died shortly after arrival. Autopsy confirmed that his death was the result of congestive heart failure. There was severe peripheral edema, the heart was enlarged and the right ventricle was dilated. Apart from congestion of the abdominal organs and the brain, autopsy revealed ascites and pericardial effusion which supported the clinical diagnosis fluid retention due to congestive heart failure.

### Comparison to controls

In **Tables 1**, **2**, we compare the four cases with CV events to a control population of 117 adults with PWS without CV events. Compared to the cases, controls had a lower BMI and more often had used growth hormone treatment during childhood. CV risk factors were also frequent in control patients. Peripheral edema was present in 31% of the controls.

**Table 1:** Patient characteristics, cardiovascular risk factors and childhood GH status of four patients with PWS with cardiovascular events, compared to 117 PWS adults without cardiovascular events.

	Age (years) ^a^	Genotype	BMI (kg/m^2^)	Type 2 diabetes mellitus	Hyper-tension	Hyperchol-esterolemia	Sleep apnea	GH during childhood
Patient 1	39	Deletion	45	Yes	Yes	Yes	Yes	No
Patient 2	37	Deletion	44	Yes	No	Yes	Yes	No
Patient 3	29	Deletion	48	No	No	No	No	No
Patient 4	32	Unknown	53	NA	Yes	No	NA	No
Control (n=117)	28 [20 – 38]	Deletion: 61 (52%) mUPD: 42 (36%) ICD: 3 (3%) Unknown: 11 (9%)	29 [26 – 35]	13 (11%)	17 (15%)	20 (17%)	17 (15%)	58 (50%)

Data presented as yes (present), no (absent) or NA (not available) for individual patients and as n (%) for control PWS adults. For controls the age and BMI are given as median [IQR].

Abbreviations: body mass index (BMI), growth hormone (GH), imprinting center defect (ICD), maternal parental disomy (mUPD). ^a^ Age at first event for cases and age at data collection for controls.

**Table 2:** Physical complaints and signs of cardiac decompensation of four patients with PWS with cardiovascular events, compared to 117 PWS adults without cardiovascular events.

	Chest pain	Peripheral edema	Orthopnea	Progression of edema	Increased NT-proBNP	Difficulty interpreting cardiac ultrasound	Signs of pulmonary hypertension ^a^
Patient 1	No	Yes	Yes	Yes	No	Yes	No
Patient 2	Yes	Yes	NA	Yes	NA	Yes	Yes
Patient 3	No	Yes	Yes	Yes	Yes	Yes	Yes
Patient 4	No	Yes	Yes	Yes	Yes	NA	Yes
Control^b^	2/91 (2%)	29/95 (31%)	1/90 (1%)	NA	NA	NA	NA

Data presented as yes (present), no (absent), NA (not available) for individual patients and as number of controls with the outcome / total number of controls for whom the outcome was known (%) for the group of control patients.

^a^ Signs of pulmonary hypertension include: right axis deviation on electrocardiogram, signs of increased systolic right ventricle pressure on echocardiogram (in absence of pulmonary valve stenosis): increased tricuspid regurgitation velocity, enlarged vena cava and/or right ventricle dilatation or hypertrophy. ^b^ Number of controls differs as a result of missing values.

**Figure 1:**
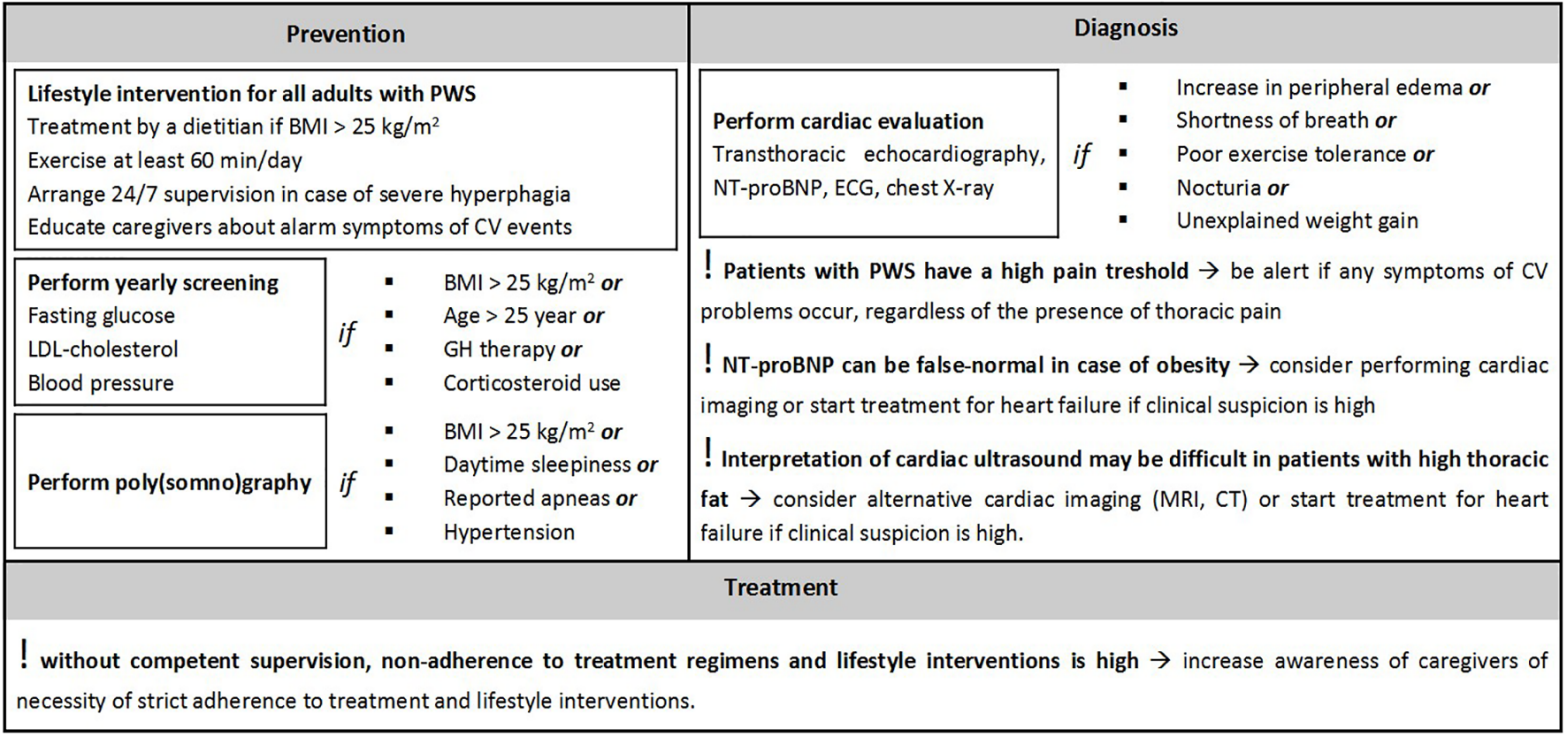
Recommendations for prevention, diagnosis and treatment of cardiovascular events in adults with Prader-Willi syndrome. Abbreviations: 24 hours a day, 7 days a week (24/7), body mass index (BMI), computed tomography (CT), cardiovascular (CV), electrocardiogram (ECG), growth hormone (GH), low-density lipoprotein (LDL), minutes (min), magnetic resonance imaging (MRI), Prader-Willi syndrome (PWS). These recommendations only consider factors related to cardiovascular disease. For the full screening protocol for prevention, see Pellikaan et al. (17).

Based on the described cases, the literature and our clinical expertise, we formulated recommendations for prevention, diagnosis, and treatment of CV events in adults with PWS, see **Figure 1**.

## Discussion

We describe the syndrome-specific challenges encountered during the diagnosis and treatment of severe CV problems, i.e. heart failure, of four adults with PWS. Diagnosis was complicated by obesity (BMI between 44 and 53 kg/m^2^) and pre-existent peripheral edema.

Pulmonary hypertension played a key role in the pathogenesis of CV disease in all patients described. Dilated right ventricle and dilated vena cava inferior, both signs of increased right heart pressure, were present in most patients. Left ventricle function was usually normal.

Factors contributing to CV disease were obesity (n=4), DM2 (n=2), hypertension (n=2), hypogonadism (n=3) and sleep apnea (n=2) (37). Remarkably, all patients had their first cardiac decompensation before the age of 40, the youngest patient being 29 years old. This is in contrast with the low prevalence of heart failure of 0.1-0.5% found in non-PWS adults in this age category (with and without overweight) (39).

CV disease in adults with PWS is caused by a complex interplay of several syndrome-specific characteristics, which eventually leads to obesity (17). Obesity is associated with systemic low-grade inflammation and oxidative stress, hypertension, hypercholesterolemia, insulin resistance and DM2, all associated with increased CV risk (40–43). Furthermore, obesity can lead to obesity-associated hypoventilation syndrome (OAHS) (44), with subsequent pulmonary hypertension and right ventricular failure, if left untreated. In addition to the obesity-related increase in CV risk, patients with PWS have an additional risk due to decreased microvascular function that is associated with the syndrome (45), as endothelium and microvessels may play an important role in the pathogenesis of heart failure (46, 47). Lastly, scoliosis is often present in patients with PWS (23). If severe, scoliosis can cause restrictive pulmonary dysfunction, pulmonary hypertension and eventually CV decompensation (48, 49).

Due to the cumulative effect of the above-mentioned mechanisms, the patients we described developed cardiac decompensation at an exceptionally young age. To prevent the development of CV disease, it is important to identify and treat CV risk factors early in life. Prevention and treatment of obesity may be complicated due to intellectual disability and hyperphagia. Therefore a multidisciplinary approach is needed. A (pediatric) endocrinologist, specialized dietitian, physiotherapist and, if needed, a behavioral expert or psychologist should work together to avoid or treat obesity. Additionally, it is essential to have adequate supervision at home to control food intake. Specialized PWS homes can be beneficial to ensure this supervision. If PWS homes are unavailable, caregivers should receive clear instructions about restricting the patient’s food intake and increasing physicial activity. Additionally, the endocrinologist should screen yearly for CV risk factors like hypertension, DM2, and hypercholesterolemia. For this screening, our previously described algorithm may be helpful (17). These measures might prevent long-term debilitating CV complications as seen in the four patients we described.

If, despite prevention, heart failure develops, the diagnosis of heart failure can be challenging in patients with PWS, especially when obesity is present. Peripheral non-pitting edema (lipedema and lymphedema) is common in PWS adults without CV morbidity (31%), which makes edema, in general, a poor marker for CV deterioration. However, in all four patients who eventually developed cardiac decompensation, edema was of the pitting kind and progressive. Therefore, an increase in peripheral pitting edema should be considered an alarm symptom and should trigger further investigation.

Apart from progressive pitting edema, all cases showed orthopnea and/or progressive shortness of breath. Although not systematically assessed, none of the patients complained of nocturia. When symptoms of heart failure like dyspnea and/or orthopnea are present, additional testing is needed to rule out cardiac problems. It should be emphasized that, in obese subjects, false negative NT-proBNP values can put physicians on the wrong track. Likewise, cardiac ultrasound can be hard to interpret due to poor imaging quality resulting from obesity (35).

Besides diagnostic challenges, it can also be challenging to initiate and maintain adequate cardiac treatment. In general, diuretics and fluid and salt restriction are essential for treatment of congestive heart failure. However, due to hyperphagia and intellectual disability, nonadherence to fluid and salt restriction is common in adults with PWS. Adherence can only be guaranteed by competent supervision, which means that 24/7 supervision is often crucial in heart failure treatment in PWS. Non-adherence to CPAP may also occur. As patients with OAHS benefit from CPAP, this is a useful intervention. However, the CPAP mask can cause anxiety, especially in case of intellectual disability. In that case, stepwise introduction of the mask (starting with a few minutes per day) and gradual increase in usage is crucial to prevent non-compliance. Also, for the acceptance of CPAP, it may be useful to involve a psychologist or behavioral expert.

### Strengths and limitations

One of the strengths of the current study is the detailed description of four cases of PWS adults with CV events. To our knowledge, we are the first to describe cases of PWS adults with CV events and to provide practical recommendations based on the similarities between these cases. A limitation of this study is that this study was retrospective and therefore some details were unknown. Moreover, the diagnostic tests were performed in different hospitals, which may have caused some variation in results of imaging and/or biochemical tests.

## Conclusion

In conclusion, the diagnostic trajectory and treatment of CV disease in adults with PWS can be extremely challenging. Peripheral edema, a reliable marker of right-sided heart failure in the general population, is frequently present in the general PWS population and is therefore not a good indicator of heart failure. Diagnosis of heart failure is further hindered by the decreased reliability of NT-proBNP levels and increased technical challenges in transthoracic echocardiography in case of obesity. To prevent doctors’ delay, it is important to inform general practitioners, ID physicians, internists and cardiologists about these diagnostic pitfalls. To prevent patient delay, it is important to inform caregivers about early signs of cardiac failure, like exercise-related shortness of breath, progression of peripheral edema and unexplained weight gain. Diagnosis and treatment can be complicated by PWS-specific behavior, non-compliance to salt and water restriction, fear of CPAP and refusal of medication. Therefore, preventive measures, diagnostics and treatment of CV disease should preferably be guided by a multidisciplinary team.

## Data availability statement

The datasets presented in this article are not readily available in order to protect the privacy of the patients participating in this study. As Prader–Willi syndrome is a rare syndrome, individual patient data could be traced back to the individual. Requests to access the datasets should be directed to Laura de Graaff, l.degraaff@erasmusmc.nl.

## Ethics statement

The studies involving human participants were reviewed and approved by the medical ethical committee of the Erasmus MC. Written informed consent was obtained from the individual(s) for the publication of any potentially identifiable images or data included in this article.

## Author contributions

Conceptualization, KP and LG. Methodology, KP and LG. Formal analysis, KP. Investigation, KP, PW and LG. Resources, LG. Writing—original draft preparation, KP and PW. Writing—review and editing, all authors. Visualization, KP. Supervision, LG and AL. Project administration, KP and LG. All authors contributed to the article and approved the submitted version.
