# Supplementary material for: What endocrinologists can do to prevent cardiovascular complications in adults with Prader-Willi syndrome: Lessons from a case series
Source: Front Endocrinol (Lausanne). 2023 Mar 24;14:1145066. doi: 10.3389/fendo.2023.1145066 (PMC10080071; doi:10.3389/fendo.2023.1145066)
Supplement: Supplementary file 1 [file Table_1.docx]

Supplementary Material

What endocrinologists can do to prevent cardiovascular complications in adults with Prader-Willi syndrome: lessons from a case series

Karlijn Pellikaan^1,2,3,4^, Paula M.H. van Weijen^1^, Anna G. W. Rosenberg^1,2,3,4^, Franciska M. E. Hoekstra^5^, Michiel Vermaak^6^, Peter H. N. Oomen^7^, Aart J. van der Lely^1^, Judith A.A.E. Cuypers^8^, Laura C. G. de Graaff^1,2,3,4*^

^1^ Department of Internal Medicine, Division of Endocrinology, Erasmus University Medical Center Rotterdam, Rotterdam, The Netherlands

^2^ Center for Adults with Rare Genetic Syndromes, Department of Internal Medicine, Division of Endocrinology, Erasmus University Medical Center Rotterdam, Rotterdam, The Netherlands

^3^ Dutch Center of Reference for Prader-Willi Syndrome, Rotterdam, The Netherlands

^4^ Academic Centre for Growth Disorders, Erasmus University Medical Center Rotterdam, Rotterdam, The Netherlands

^5^ Department of Internal Medicine, Reinier de Graaf hospital, Delft, the Netherlands

^6^ Novicare, Best, the Netherlands

^7^ Department of Internal Medicine, Medical Center Leeuwarden, Leeuwarden, the Netherlands

^8^ Department of Cardiology, Erasmus University Medical Center Rotterdam, Rotterdam, The Netherlands

*** Correspondence:**Laura de Graaff, MD, PhD

E-mail l.degraaff@erasmusmc.nl

# Supplementary Figures and Tables

**Table S1.** Details of patient 1 at time of cardiovascular event

| - **Medical history** - Hypertension: yes - Myocardial infarction: no - Cerebrovascular accident: no - Diabetes mellitus: yes - Hypercholesterolemia: yes - Use of medication: gemfibrozil, hydrochlorothiazide, metformin, insulin, testosterone replacement therapy (tostran) |
| --- |
| - **Family history cardiovascular disease** - Hypertension: yes - Myocardial infarction: no - Cerebrovascular accident: no |
| - **Anamnesis** - Dyspnea at rest: yes - Dyspnea during exercise: yes - Orthopnea: yes - Palpitations: no - Angina pectoris: no - Smoking: yes |
| - **Physical examination** - Respiratory rate: up to 40 breaths per minute  saturation: 90% - Blood pressure: 166/ 86 mmHg - Heart sounds: soft cardiac sounds - Heart rate: 88 beats per minute, regular - Pulmonary auscultation: rhonchi all fields, vesicular breath sounds both sides, extended wheezing expiration all fields - Weight: 134 kg - Height: 172 cm - BMI: 45 kg/m^2^ - Edema: yes, pitting edema of the legs |
| - **Laboratory measurements:** - Glucose: 6.3 mmol/L - Urea: 4.4 mmol/L - Creatinine: 51 µmol/L - eGFR: 129 mL/min - Sodium: 140 mmol/L - Potassium: 4.3 mmol/L - Calcium: 2.34 mmol/L - Albumin: 40 g/L - ASAT: 44 U/L - ALAT: 69 U/L - Lactate dehydrogenase: 230 U/L - GGT: 49 U/L - Alkaline phosphatase: 131 U/L - Total bilirubin: 4 µmol/L - LDL-cholesterol: 2.97 mmol/L - Hemoglobin: 8.2 mmol/L - Hematocrit: 0.43 L/L - MCV: 87 fL - RDW: 14.7 % - Thrombocytes: 168 10^9/L - Leukocytes: 5.4 10^9/L - TSH: 0.274 mU/L - FreeT4: 22.7 pmol/L - NT-proBNP: 20 pmol/L - CRP: 30 mg/L |
| - **Electrocardiogram:** - Sinus rhythm, normal conduction times, no ST elevation or depressions, normal R wave progression |
| - **Chest X-ray:** - Slightly blurred left heart margin |
| - **Echocardiography:** Transthoracic: normal systolic left and right ventricle function, slim right ventricle, normal dimension left atria, no signs of diastolic dysfunction, right ventricle pressure not measurable**.** |
| - **MRI:** No cardial cause for pulmonal hypertension, no systolic ventricle dysfunction and diastolic values are normal. Atria not dilated which pleats against increased diastolic pressure left. Right heart is slim and does not show any signs of pulmonic hypertension |
| - **CT angiography coronary arteries:** Focal calcifications left anterior descending artery, right coronary artery and intermediary artery with local stenosis up to 50 %. Calcium score 146. |
| - **Embletta:**   Apnoea–Hypopnoea Index: 14 |

**Table S2.** Details of patient 2 at time of cardiovascular event

| - **Medical history** - Hypertension: yes - Myocardial infarction: no - Cerebrovascular accident: no - Diabetes mellitus: yes - Hypercholesterolemia: yes |
| --- |
| - **Family history cardiovascular disease** - Hypertension: yes - Myocardial infarction: yes |
| - **Anamnesis** - Dyspnea at rest: yes - Dyspnea during exercise: yes - Orthopnea: yes - Palpitations: no - Angina pectoris: no - Smoking: no |
| - **Physical examination** - Weight: 110.8 kg - Height: 158 cm - BMI: 44 kg/m^2^ - Edema: yes (pitting edema of the legs) |
| - **Electrocardiogram:** - Negative T waves in leads V1-3 |
| - **Echocardiography:** - Full tricuspid valve insufficiency with signs of elevated right ventricular pressure and a dilated vena cava inferior report |

**Table S3.** Details of patient 3 at time of cardiovascular event

| - **Use of medication** - Hypertension: yes - Use of medication before hospitalization: Thyrax 137.5 ug, oral contraceptive pill, movicolon, vitamin D - Use of medication after discharge:   Amlodipine 10 mg 2 times a day  Vitamin D3 800 IE once a day  Hydrocortisone/miconazol 10/20 mg/g cream cutaneous  Levetiracetam 100 mg/ ml drink 760 mg 2 times a day  Levothyroxine 125 mcg once a day  Metoprolol 25 mg once a day  Perindopril 8 mg once a day  Spironolactone 12.5 mg once a day |
| --- |
| - **Anamnesis** - Dyspnea at rest: yes - Dyspnea during exercise: yes - Orthopnea: yes |
| - **Physical examination** - Heart rate: 73 beats per minute, regular - Weight: 115 kg - Height: 158 cm - BMI: 48 kg/m^2^ - Edema: yes (pitting edema of the legs) |
| - **Laboratory measurements:** - Thrombocytes: increased - Leukocytes: increased - NT-proBNP: 99 pmol/L - Sodium: 134 mmol/L |
| - **Electrocardiogram:** - Sinus rhythm, 73 /min, negative T in lead V1-3 |
| - **X ray chest**: - Enlarged heart, no signs of congestion |
| - **Echocardiography:** - Normal systolic left ventricle function, left ventricle ejection fraction 55%, dilated right ventricle, normal systolic right ventricle function. Mild tricuspid valve insufficiency. Right pressure not measurable. Vena cava inferior diameter 2.2 cm. D shaped septum. |

**Table S4.** Details of patient 4 at time of cardiovascular event

| - **Use of Medication** - Use of medication during hospitalization: Burinex 5mg 2 times a day  Amoxicillin 500 mg 4 times a day Ciproxin 500 mg 2 times a day Hydrocortisone 10-5-5 mg - Medication at discharge: - Vitamin D 800 IE once a day - Lisinopril 2.5 mg once a day |
| --- |
| - **Anamnesis** - Dyspnea at rest: yes - Dyspnea during exercise: yes - Orthopnea: yes |
| - **Physical examination** - Respiratory rate: 28 per minute, saturation: 76% - Blood pressure: 139 / 108 mmHg - Heart rate: 120 beats per minute, regular - BMI: 53 kg/m^2^ - Edema: yes, pitting edema of the legs |
| - **Laboratory measurements:** - Glucose: 6.7 mmol/L - Urea: 7.3 mmol/L - Creatinine: 51µmol/L - eGFR: 135 mL/min - Sodium: 140 mmol/L - Potassium: 4.2 mmol/L - ASAT: 35 U/L - ALAT: 62 U/L - Lactate dehydrogenase: 274 U/L - Triglycerides: 0.6 g/L - Total cholesterol: 3.0 mmol/L - HDL-cholesterol: 0.85 mmol/L - LDL-cholesterol: 1.90 mmol/L - Hemoglobin: 8.0 mmol/L - Hematocrit: 0.42 L/L - MCV: 85 fL - Thrombocytes: 264 10^9/L - Leukocytes: 8.3 10^9/L - TSH: 2.28 mU/L - NT-proBNP: 230 pmol/L - CRP: 94 mg/L - *Urine:* - Protein: + |
| - **Electrocardiogram** (performed a few weeks before hospitalization )**:** - Sinus rhythm, negative T waves in leads III, aVF and V1-5, left axis cardiac deviation and right bundle branch block. |
| - **Autopsy:** Enlarged heart, ventricles slightly contracted, right ventricle dilation. |
